# Oncological Follow-up Strategies for Testicular Germ Cell Tumours: A Narrative Review

**DOI:** 10.1016/j.euros.2022.08.014

**Published:** 2022-09-07

**Authors:** Ernest Kaufmann, Luca Antonelli, Peter Albers, Clint Cary, Silke Gillessen Sommer, Axel Heidenreich, Christoph Oing, Jan Oldenburg, Phillip Martin Pierorazio, Andrew J. Stephenson, Christian Daniel Fankhauser

**Affiliations:** a University of Zurich, Zurich, Switzerland; b Department of Urology, Luzerner Kantonssspital, Lucerne, Switzerland; c Department of Urology, Medical Faculty, Heinrich-Heine University, Düsseldorf, Germany; d Department of Urology, Indiana University School of Medicine, Indianapolis, IN, USA; e Oncology Institute of Southern Switzerland, Bellinzona, Switzerland; f Faculty of Biosciences, USI University, Lugano, Switzerland; g Department of Urology, University Hospital Cologne, Cologne, Germany; h Department of Urology, Medical University of Vienna, Vienna, Austria; i Sir Bobby Robson Cancer Trials Research Centre, Department of Cancer Services, Freeman Hospital, Newcastle upon Tyne Hospitals NHS Foundation Trust, Newcastle upon Tyne, UK; j Department of Oncology, Akershus University Hospital and Medical Faculty of University of Oslo, Oslo, Norway; k The James Buchanan Brady Urological Institute and Department of Urology, Johns Hopkins University School of Medicine, Baltimore, MD, USA; l Department of Urology, Rush University Medical Group, Chicago, IL, USA

**Keywords:** Testis, Germ cell tumour, Relapse, Follow-up, Guidelines, Serum markers, Cross-sectional imaging

## Abstract

**Context:**

The aim of this review is to describe the proportion of testicular germ cell tumours (tGCTs) with recurrence, and the timing and anatomical sites of relapse across different disease stages and after different treatment options. We summarise published follow-up protocols and discuss current and future developments to personalise follow-up for patients with tGCT.

**Evidence acquisition:**

A systematic literature search was conducted and current guidelines and selected institutional follow-up protocols were reviewed.

**Evidence synthesis:**

Of 302 publications, we screened 68 full texts and included 29 studies; 22 of these were retrospective and seven were prospective in nature, contributing data for 20 570 patients. The number of patients included per study ranged from 119 to 2483. We compared the guideline follow-up protocols of the European Society for Medical Oncology, European Association of Urology, National Comprehensive Cancer Network, and American Urological Association, as well as institutional follow-up protocols. The protocols differed in terms of the number, time points, and type of follow-up investigations.

**Conclusions:**

Future research should assess how tGCT can be followed to ensure high adherence, define the role of miR-371a-3p microRNA during follow-up, and develop follow-up protocols after curative treatment in the metastatic setting.

**Patient summary:**

In this review of follow-up protocols for men with testis cancer, we observed different recommendations and discuss future research areas to improve follow-up for these patients.

## Take Home Message

Testicular germ cell tumour is a rare and highly curable malignancy with excellent survival rates. Guideline and institutional protocols differ in the modality, number, and timing for follow-up investigations and can lead to suboptimal care of survivors. More prospective trials are needed to assess how patients can be followed to ensure high adherence, define the role of miR-371a-3p in follow-up, and develop protocols for follow-up after curative treatment in the metastatic setting.

## 1 Introduction

Testicular germ cell tumour (tGCT) is a rare malignancy but is also the most common solid tumour among men aged 15–40 yr. Fortunately, approximately 95% of patients with tGCT overall and 80% with metastatic disease can be cured [1]. Although tGCT is a rare cancer, the high cure rate leads to a large cohort of long-term survivors. After cancer treatment is completed, attention turns to follow-up strategies, including regular measurement of the serum tumour markers (STMs) α-fetoprotein (AFP), human chorionic gonadotropin (hCG), and lactate dehydrogenase (LDH). As these STMs detect only approximately 60% of nonseminoma and 5% of seminoma recurrences [2], additional cross-sectional imaging (computed tomography [CT] or magnetic resonance imaging [MRI]) is required during follow-up.

Metastatic relapse in men with localised disease is classified according to the International Germ Cell Cancer Collaborative Group (IGCCCG), and men in the good prognosis group require only three cycles instead of the four cycles of cisplatin-based combination chemotherapy required for men in the intermediate or poor prognosis group. An important aim of follow-up investigations is therefore to detect disease recurrence at an early stage, while the disease is classified in the IGCCCG good prognosis group, in which case less treatment is required for cure and the prognosis is better. The aim of this review was to summarise published follow-up protocols and discuss current and future developments to personalise the oncological follow-up for patients with tGCT.

## 2 Evidence acquisition

We performed a literature search on March 28, 2022 to summarise follow-up protocols published in the literature (Supplementary material) and in current guidelines (European Society for Medical Oncology [ESMO], European Association of Urology [EAU], National Comprehensive Cancer Network [NCCN], American Urological Association [AUA]), as well as the follow-up protocols of published networks or institutions, including the Swedish and Norwegian Testicular Cancer Group (SWENOTECA), Swiss Austrian German Testicular Cancer Cohort Study, and Princess Margaret Cancer Center (Toronto, Canada). Non-English literature and publications before 1990 were excluded. The reference lists of the publications selected were manually screened to identify further publications, and duplicate articles were filtered using the “close match function” in Endnote and manual deduplication. Two investigators (E.K. and L.A.) screened all the titles, abstracts, and full texts for appropriateness. Data from the same studies that appeared in multiple publications were only considered once. Finally, only published manuscripts for cohorts with a minimum of 100 men were included. Any discrepancies were resolved by a third investigator (C.D.F.).

## 3 Evidence synthesis

We identified 302 publications that met the initial search criteria and proceeded with a title and abstract screening review of 68 full texts (Supplementary Fig. 1). In total, 29 studies were selected for inclusion, of which 22 were retrospective and seven were prospective in nature (Supplementary Table 1). The 29 studies included a total of 20 570 patients with any stage of tGCT. The number of patients included per study ranged from 119 to 2483.

### 3.1 Oncological follow-up

#### 3.1.1 Stage I seminoma

For clinical stage I seminoma, both surveillance and adjuvant chemotherapy are valid options after orchiectomy, as overall survival for these patients is excellent with both treatments and does not significantly differ between them [3], [4]. Approximately 3–20% of these patients experience relapse at a median of 14–21 mo after orchiectomy [2], [3], [4]. After surveillance, relapse is observed in 6–20% of cases, whereas relapse after adjuvant treatment is observed in 3–6% [2], [3], [4], [5]. Approximately 75–95% of all recurrences can be observed within the first 2–3 yr and >95% within 5 yr [6], [2], [7], [8], [9]. For nearly 90% of relapses, recurrence is observed in the retroperitoneum, for which an abdominal CT scan is the most common modality for detection [2], [5], [10]. By contrast, recurrence detected via clinical examination, chest X-ray, or STMs (bHCG, AFP, or LDH) alone is observed in 0–5%, 0%, and 5–10% (bHCG 3–11%, AFP 0–2%) of cases, respectively [2], [7], [9], [11]. Although seminomatous GCT by definition does not produce AFP, this marker is assessed during follow-up as a few patients initially diagnosed with seminoma may experience recurrence with an AFP-producing nonseminomatous GCT. Isolated pelvic metastases are observed in <1% of patients overall and 5% of patients with recurrence [5], [10], [12], [13].

The aforementioned site-specific and modality-specific recurrence patterns have resulted in recommended follow-up that includes abdominal CT scans every 6 mo in the first 2–3 yr and every 12 mo in years 4 and 5 (Table 1). Those recommendations are likely to change after the recent publication of data from the TRrial of Imaging and Surveillance in Seminoma Testis (TRISST) [11]. This randomised trial demonstrated the noninferiority of MRI compared to CT during follow-up for patients with stage I seminoma and the noninferiority of three versus seven radiological assessments using cross-sectional imaging. Although there was an absolute increase of 2.5% in events with three versus seven scans, the 5-yr survival was excellent at 99% in all arms. In summary, the TRISST data will increase the use of MRI and help to reduce the frequency of imaging [11]. Imaging of the chest is not recommended in any guideline or institutional follow-up protocol. STMs are recommended in all guidelines except for the NCCN, AUA, and Princess Margaret Cancer Center guidelines, which state that STM measurement is optional (Table 1).

**Table 1:** Comparison of follow-up schedules including investigations and their intervals for patients with stage I testicular germ cell tumour stratified by histology and adjuvant treatment received^a^

Histology and treatment	STM/CTAP/CXR/CTC interval (mo)					
	EAU/ESMO	NCCN	SWENOTECA	SAGTCCS	Toronto	AUA
**Seminoma**						
AS						
Year 1	6/6/0/0	(3–6)/4–6 & 12/b/b	6/6^c^/0/0	3/6/12/0	d/6/0/0	d/4–6/0/0
Year 2	6/6/0/0	(6)/6/b/b	6/6^c^/0/0	3/6/0/0	d/6/0/0	d/4–6/0/0
Year 3	6/12/0/0	(6–12)/6–12/b/b	6/12^c/^0/0	6/12/0/0	d/6/0/0	d/6–12/0/0
Year 4	12/0/0/0	(12)/12–24/b/b	6/12^c^/0/0	6/0/0/0	d/12/0/0	d/6–12/0/0
Year 5	12/12/0/0	(12)/12–24/b/b	12/12^c^/0/0	6/12/0/0	d/12/0/0	d/6–12/0/0
Year >5					d/12/0 (until y9), no CT in y6 & y8, CXR at y9	
Adjuvant CBP						
Year 1	Same as for AS	(6–12)/12/b/b	6/6^c/^0/0	Same as for AS	Same as for AS	Same as for AS
Year 2		(6–12)/12/b/b	6/6^c/^0/0			
Year 3		(12)/12/b/b	6/12^c/^0/0			
Year 4		(12)/0/b/b	6/12^c^/0/0			
Year 5		(12)/0/b/b	12/12^c^/0/0			
Year >5			STM/MRI at 7/10 yr			
Adjuvant RT						
Year 1	Same as for AS	(6–12)/12/b/b	6/0^c^/0/0	Same as for AS	Same as for AS	Same as for AS
Year 2		(6–12)/12/b/b	6/12^c^/0/0			
Year 3		(12)/12/b/b	6/0^c^/0/0			
Year 4		(12)/0/b/b	6/0^c^/0/0			
Year 5		(12)/0/b/b	12/12^c^/0/0			
Year >5						
**Nonseminoma**						
AS without LVI						
Year 1	3/6/6/0	2/4–6/at 4 & 12/b	3/6^c/^0/0	2/at 4 & 12/4/0	2 (+m1)/4/0/4	2–3/3–6/3–6/0
Year 2	3/12/6/0	3/6/12/b	3/6^c^/0/0	3/6/6/0	2/12/0/12	2–4/4–12/4–12/0
Year 3	6/12/0/0	4–6/12/12/b	6/12^c^/0/0	6/12/12/0	4/0/0/0	4–6/12/12/0
Year 4	6–12/0/0/0	6/b/12/b	6/12^c^/0/0	6/0/0/0	6/0/0/0	6–12/12–24/12–24/0
Year 5	(6–)12/12/0/0	12/b/b/b	6/12^c^/0/0	6/12/12/0	12/12/0/12	6–12/12–24/12–24/0
Year >5						
AS with LVI						
Year 1	Same as for AS	2/4/4/b	2/6^c^/0/0	2/4/4/0	2 (+ m1)/4/0/4	Imaging at shorter intervals than without LVI (no specific schedule)
Year 2	without LVI	3/4–6/12/b	3/6^c^/0/0	3/12/6/0	2/12/0/12	
Year 3		4–6/6/6/b	6/12^c^/0/0	6/12/12/0	4/0/0/0	
Year 4		6/12/12/b	6/12^c^/0/0	6/0/0/0	6/0/0/0	
Year 5		12/b/b/b	6/12^c^/0/0	6/12/12/0	12/12/0/12	
Year >5						
Adjuvant 1× BEP						
Year 1	3/6/6–12/0	3/12/6–12/b	3/12^c^/0/0	3/6/6/0	Not available	Not available
Year 2	3/12/12/0	3/12/12/b	3/12^c^/0/0	3/12/12/0		
Year 3	6/12/12/0	6/0/0/0	6/12^c^/0/0	6/12/12/0		
Year 4	6/0/0/0	6/0/0/0	6/0^c^/0/0	6/0/0/0		
Year 5	6/12/12/0	12/0/0/0	6/12^c^/0/0	6/12/12/0		
Year >5						

STM = serum tumour markers; CTAP = abdominopelvic computed tomography; CXR = chest X-ray; CTC = chest CT; EAU = European Association of Urology; ESMO = European Society for Medical Oncology; NCCN = National Comprehensive Cancer Network; SWENOTECA = Swedish and Norwegian Testicular Cancer Group; SAGTCCS = Swiss Austrian German Testicular Cancer Cohort Study; AUA = American Urological Association; AS = active surveillance; RT = radiotherapy; LVI = lymphovascular invasion; CBP = carboplatin; BEP = bleomycin, etoposide, and cisplatin; y9 = year 9; m1 = month 1; MRI = magnetic resonance imaging (abdominopelvic).

a Optional investigations are in parentheses.

b As clinically indicated (CXR when CTAP is obtained; consider CTC instead of CXR in symptomatic patients).

c SWENOTECA explicitly recommends MRI instead of CT.

d No routine STM measurements recommended in the AUA and Toronto guidelines for seminoma (for the AUA, only to be considered if elevated bHCG before orchiectomy or when clinically indicated).

There is a rationale for personalising follow-up protocols on the basis of risk factors for recurrence [14] or the use of adjuvant treatment [3], [10]. Administration of adjuvant carboplatin decreases the risk of relapse but does not change the site or timing of relapse [5], which has resulted in less stringent follow-up protocols from the NCCN and SWENOTECA. SWENOTECA recommends only two MRI scans of the abdomen after 2 and 5 yr, while the NCCN advises yearly CT scans in the first 3 yr. By contrast, the ESMO guidelines do not provide a treatment-tailored follow-up protocol after adjuvant carboplatin and recommend the same schedules for patients with and without adjuvant treatment.

Because isolated pelvic metastases are rare, some authors have suggested omission of a pelvic scan, which would decrease the cumulative radiation exposure [5], [10], [12], [13]. However, the question of whether the pelvis should be included in scans may become less relevant if MRI is used instead of CT because of the recently published TRISST data. In addition, if previous results for the novel biomarker miR-371a-3p are confirmed in large prospective cohorts, the number of scans may decrease even further [15].

#### 3.1.2 Stage I nonseminoma

For stage I nonseminoma, both surveillance and adjuvant therapy (one cycle of bleomycin, etoposide, and cisplatin, or retroperitoneal lymph-node dissection [RPLND]) are valid options after orchiectomy; approximately 80% of patients on surveillance are cured and will not need further treatment [2]. During surveillance, relapse is observed in 15–30% of patients with stage I nonseminoma at a median of 5–7 mo after orchiectomy [2], [16], [17]. Among men without lymphovascular invasion (LVI), relapse occurs in of 10–20% these cases after median follow-up of 8–21 mo, whereas relapse occurs in 40–60% of cases with LVI after median follow-up of 4–10 mo [2], [17].

Recurrences are detected in 80% of stage I nonseminoma cases within the first year and 90% (95% in LVI^+^, 89% in LVI^−^) within the first 2 yr. After the second year (late and very late relapses) 6% of all recurrences are detected and after the third year it is only 1% [2], [16]. The most common site of recurrence is the retroperitoneum (approx. 50–65%); 15–20% of recurrences occur in the chest and are rarely detected by X-ray (2%) but more precisely by a CT [2], [18], [19]. STM measurement detects recurrence in 60–65% of cases (bHCG 44%, AFP 44%), whereas clinical examination detects recurrence in <1% [16], [18]. In a randomised trial by Rustin et al [20], there was no difference in IGCCCG prognosis group at recurrence between men randomised to either CT scans of the abdomen and chest at 3 and 12 mo or at 3, 6, 9, 12, and 24 mo. Similar to seminoma, isolated pelvic metastases can only be detected in 5% of relapse cases [12], [13], [16].

Use of CT scans of the chest remains controversial in the literature and differs among recommendations. Relapse of stage I nonseminoma is rarely limited to the chest, and recurrence can often be detected via abdominal CT and/or STMs [2], [21]. In the randomised trial by Rustin et al. [20], only five of 247 cases (2%) showed STM–negative recurrence in the chest with no other sites involved; this finding is in line with retrospective studies [18], [22], [23], [24].

In comparison to stage I seminoma, there is an even stronger rationale for personalising follow-up protocols for stage I nonseminoma on the basis of pathology and adjuvant treatment options. Nearly all the protocols recommend more intense follow-up with STM measurement and imaging for patients on surveillance who had LVI with the primary testicular tumour. The somewhat inconsistent recommendations include a high number of chest X-rays according to the EAU/ESMO and more abdominal CT scans according to the NCCN guideline. The difference in chest imaging between the SWENOTECA protocols (no chest imaging) and the Toronto group (five chest CTs) during follow-up is remarkable [25].

As with stage I seminoma, isolated pelvic metastases with stage I nonseminoma are rare, and some authors have thus suggested omitting a pelvic scan, which would decrease cumulative radiation exposure [5], [10], [12], [13]. However, the question of whether the pelvis should be included in scans may become less relevant if MRI is used instead of CT. Although no randomised controlled trial has compared MRI and CT in stage I nonseminoma, similar diagnostic accuracy can be assumed on the basis of retrospective study from Denmark describing the use of abdominal MRI in 235 men with nonseminoma and promising sensitivity and specificity of >93% [19]. As a next step, the number of scans may be further decreased if previous results for the novel biomarker miRNA-371 are confirmed in large prospective cohorts [15].

#### 3.1.3 Metastatic seminoma

For metastatic seminoma, chemotherapy and radiotherapy are treatment options. After first-line chemotherapy, 30% of patients with metastatic seminoma experience a complete clinical response (normalisation of STMs and complete radiographic resolution), while 60% achieve a favourable response (normalisation of STMs but radiographic residual disease) [26]. Resection of residual masses is rarely performed for seminoma because seminomas almost exclusively contain necrosis, especially if they are smaller than 3 cm. In patients with residual tumours of >3 cm, current guidelines recommend performing ^18^F-fluorodeoxyglucose (^18^F-FDG) positron emission tomography (PET)-CT at 6–8 wk after the end of systemic treatment [27]. Owing to the limited positive predictive value of ^18^F-FDG PET-CT in this setting, optimal management of masses positive on ^18^F-FDG PET-CT remains disputable and may well only lead to closer follow-up schedules rather than immediate initiation of salvage treatment, as high-level evidence is lacking [28]. By contrast, the negative predictive value of PET negativity for residual masses is quite high and may help in easing follow-up schedules and patient distress [29].

After first-line chemotherapy for advanced seminoma, <20% of patients experience relapse at a median of 9 mo. According to the IGCCCG-Update Consortium from 2021, 89% of patients relapse in good prognosis and 79% in intermediate prognosis group [30]. As these numbers also include patients dying and progressing during first-line treatment, the real number may be lower [31], [32]. Recurrence is observed in the retroperitoneum in 80–90% of cases or in the chest in approximately 10% [31], [32].

Therefore, current follow-up protocols recommend abdominal CT scans every 6–12 mo during the first 5 yr, together with STM measurement (Table 2). Follow-up of the chest differs among protocols. For example, because relapse is rarely limited to the chest, the SWENOTECA group recommends no chest follow-up at all. Moreover, STM measurement is recommended in all guidelines except for the NCCN, which treats this as optional.

**Table 2:** Comparison of different follow-up schedules including investigations and their intervals for patients with metastatic testicular germ cell tumour stratified by histology^a^

Histology	STM/CTAP/CXR/CTC interval (mo)			
	EAU/ESMO	NCCN	SWENOTECA	SAGTCCS
**Seminoma**				
Year 1	3/6–12/6–12/6–12^b^	(3)/at 3 & 9 or 12/6/b	6/6^d^/0/0	3/6/6/12^b^
Year 2	3/12/12/12^b^	(6)/12/6/b	6/6^d^/0/0	3/12/12/12^b^
Year 3	6/12/12/0	(6)/12/0/0	6/12^d^/0/0	6/12/12/12^b^
Year 4	6/0/0/0	(6)/c/0/0	6/12^d^/0/0	6/0/0/0
Year 5	6/12/12/12^b^	(6)/c/0/0	12/12^d^/0/0	6/12/12/12^b^
Year >5				
**Nonseminoma**				
Year 1	Same as for seminoma	2/6/6/b	2–3/6^b^/6/0	3/6/6/12^b^
Year 2		3/6–12/6/b	3/6^d^/6/0	3/12/12/12^b^
Year 3		6/12/(12)/b	6/12^d^/12/0	6/12/12/12^b^
Year 4		6/b/(12)/b	6/12^d^/12/0	6/0/0/0
Year 5		6/b/0/0 (annual STM y5–y10)	6/12^d^/12/0	6/12/12/12^b^
Year >5				

STM = serum tumour markers; CTAP = abdominopelvic computed tomography; CXR = chest X-ray; CTC = chest CT; EAU = European Association of Urology; ESMO = European Society for Medical Oncology; NCCN = National Comprehensive Cancer Network; SWENOTECA = Swedish and Norwegian Testicular Cancer Group; SAGTCCS = Swiss Austrian German Testicular Cancer Cohort Study; y5 = year 5.

a Optional investigations are in parentheses.

b CTC in cases with pulmonary metastases at diagnosis (for NCCN: CTC instead of CXR in symptomatic patients or supradiaphragmatic disease at diagnosis).

c As clinically indicated.

d SWENOTECA explicitly recommends magnetic resonance imaging instead of CT.

The NCCN guidelines also provide distinct follow-up recommendations for clinical stage IIA and nonbulky stage IIB versus bulky stages IIB, IIC, and III metastatic seminoma. For stage IIA and nonbulky stage IIB, STM measurement is optional, whereas for advanced stages of the disease, STM measurements are recommended ever 2–3 mo in the first 2 yr and every 6 mo thereafter. Chest X-rays are also recommended every 2–3 mo in years 1–2 and then annually until year 5. For stages IIA and IIB, chest X-rays are only performed at 6-mo intervals for the first 2 yr. In this stage, only four CT scans are performed in the first 3 yr (three CT scans in the first 2 yr), whereas for advanced stages of the disease, seven CT scans are recommended up to year 4 (five CT scans in the first 2 yr).

#### 3.1.4 Stage II nonseminoma

For stage II nonseminoma negative for STMs, both RPLND and chemotherapy are valid options. After primary RPLND without additive chemotherapy, 12–40% of patients experience relapse, whereas only 0–4% of patients experience relapse after RPLND and additive chemotherapy (Neuenschwander et al, unpublished). The only guideline specifying a follow-up protocol after primary RPLND is the NCCN: after primary RPLND without additive chemotherapy, several measurements of serum tumours markers are recommended in the first 5 yr (Table 2). Similarly, five to ten chest X-rays and annual imaging are recommended in the first 2 yr and until year 5, respectively. Furthermore, abdominal CT is recommended every 3–4 mo in the first 2 yr. After primary RPLND and additive chemotherapy, the NCCN follow-up schedule is less intense: STMs are only measured at 6-mo interval in the first 2 yr and at 12-mo intervals thereafter. There are only six chest X-rays in the whole follow-up period up to year 5, and one CT scan 4 mo after primary RPLND. Further scans are only performed if clinically indicated.

#### 3.1.5 Metastatic nonseminoma after first-line chemotherapy

Among men with nonseminomatous tGCT and a complete response to first-line chemotherapy, approximately 20% experience relapse at a median of 3 mo. Recurrence occurs within the first 2 yr in nearly all patients; recurrence is observed in the retroperitoneum (33%) and pelvis (25%), where it can be detected via abdominopelvic CT, and in the lungs (33%). STM measurement can detect recurrence in 75% of patients (bHCG 42%, AFP 8%, LDH 25%). In 25% of relapse cases, STMs are negative [33]. Therefore, follow-up schedules recommend STM measurements every 2–3 mo and abdominal CT at 6–12-mo intervals in the first 2 yr. In addition, chest X-rays are recommended for routine follow-up, and chest CTs in cases with symptoms or pulmonary/supradiaphragmatic metastases at diagnosis.

#### 3.1.6 Late relapse

More recent publications have defines relapses in the first 2 yr, after 2–5 yr, and after 5 yr as early relapse (ER), late relapse (LR), and very late relapse (VLR), respectively [34]. Only a maximum of 3% of all patients (with and without adjuvant treatment) present with LR or VLR, with median relapse times between 5 and 9 yr reported [35], [36], [37]. The main sites are the retroperitoneum (50%) and the lungs (35%). As follow-up is often limited to 5 yr, VLRs (>5 yr) are often not detected via follow-up investigations, including cross-sectional imaging (CT, chest X-ray <40%) and STM measurement (20–60%) but via clinical symptoms such as back or abdominal pain (30–65%) [35], [36], [37].

To detect LRs, the NCCN advises annual STM measurement after 5 yr up to year 10. SWENOTECA recommends STM measurement and MRI of the retroperitoneum after 7 and 10 yr for patients with intermediate or poor prognosis, residual tumours, teratoma in testis without RPLND, or teratoma in residual tumour resections.

### 3.2 Discussion

We investigated what proportion of tGCT cases recur and described the timing and anatomical sites of relapse across different disease stages and after different treatment options. Those key statistical figures are the basis for follow-up protocols, and only a limited number of prospective trials comparing different follow-up protocols are available. In addition, we summarised published follow-up protocols and discuss current and future developments to personalise follow-up for patients with tGCT. In summary, we believe that future research to improve tGCT follow-up should focus on (1) determining how patients with tGCT can be monitored to ensure high adherence to follow-up protocols, (2) defining the role of miR-371a-3p as a new and potentially more accurate biomarker during follow-up, and (3) developing new follow-up protocols for the metastatic setting.

To decrease cumulative radiation exposure in this young patient population mainly presenting with localised disease, the number of follow-up scans recommended has decreased over time [11], [38], [39]. Guideline recommendations and institutional follow-up protocols still differ regarding the modality, number of investigations, and timing for follow-up. Health care–specific differences can, to some extent, explain this variance, whereas other differences are likely to be explained by a lack of prospective data to provide a personalised follow-up schedule; this lack of data has prevented any global consensus regarding follow-up protocols. Given the recent data from TRISST [11], it is our opinion that MRI instead of CT should be regarded as an attractive imaging tool during follow-up if available and affordable. Another point in favour of MRI is the elimination of potential allergic reactions to iodine-containing contrast media. Gadolinium-based contrast agents show a very low risk of adverse events such as nephrogenic systemic fibrosis [40]. By contrast, we want to emphasise that ^18^F-FDG PET-CT should only be used in highly selected cases, such as men with pure seminomatous postchemotherapy residual disease. In general, most of the follow-up protocols published have proven to be safe, meaning that for most men any recurrence can be detected while they are still in the IGCCCG good prognosis group. This suggests that all the above-mentioned protocols seem to be oncologically safe as long as there is adherence to close follow-up.

Low adherence to published protocols represents a major challenge. Nowadays, patients with tGCT are geographically very mobile; this could explain the low adherence to follow-up protocols, possibly leading to a high proportion of relapses detected at an advanced cancer stage requiring intense treatment, which can lead to treatment-related toxicity [39], [41], [42], [43]. We are not aware of any ongoing clinical trials to assess follow-up protocols, but future studies should involve implementation science. Studies such as the WATChmAN trial (Web-based virtual Testicular CANcer clinic) have the potential to ensure complete follow-up despite being performed virtually (NCT03360994).

To personalise follow-up protocols, a correct histopathological diagnosis of the presence or absence of rete testis or LVI is important. Such a personalised approach might be limited by the considerable inter-reader discrepancy of 20–30% for these variables, as suggested in the literature [44], [45], [46]. Therefore, centralised expert pathology review should be considered, as this is an important step in improving follow-up for tGCT.

Studies are needed to indicate how measurement of circulating miR-371a-3p levels could be used to detect relapse [47]. Previous studies have found (1) promising diagnostic accuracy of miR-371a-3p in the preorchiectomy setting [48] and at the time of macroscopic recurrence [48], [49]; (2) a short half-life for miR-371a-3p [48]; and (3) high diagnostic accuracy of miR-371a-3p in identifying stage I cancer with micrometastatic disease that is not yet visible [50]. However, in a cohort of 151 men, postorchiectomy miR-371a-3p levels were not associated with disease relapse during follow-up [51]. The accuracy of a real-time quantitative reverse transcription-polymerase chain reaction protocol without preamplification in this setting was confirmed, with disease recurrence identified at a median of 2 mo (range 0–5) earlier than via standard investigations [15].

Findings for two large cohorts (NCT04435756, NCT04914026) have not yet been reported, and may confirm the accuracy of circulating miR-371a-3p measurements during follow-up and decrease the number of regular cross-sectional imaging investigations during surveillance. These changes would in turn reduce health care costs and radiation exposure. In particular, men with pure seminomatous tGCT might only require cross-sectional imaging when circulating miR-371a-3p levels increase. At present, regular imaging cannot be entirely omitted for men with nonseminomatous GCT, as recurrences with pure teratomatous GCT are not detected by miR-371a-3p measurement; however, future follow-up protocols will recommend a lower number of cross-sectional scans and therefore reduce costs and exposure to ionising radiation.

Because most GCT relapses occur within 2 yr, 2-yr disease-free survival was historically considered to be equivalent to cure, and later relapse was described as a distinct clinical entity. For men with localised disease at initial presentation and without adjuvant treatment, ER, LR, and VLR are observed in 15%, 5%, and 1% of seminoma cases, and 30%, 2%, and 1% of nonseminoma cases, respectively [34]. Notably, men with LR/VLR after orchiectomy are less likely to show elevated STMs in comparison to men with ER, but a comparable metastatic pattern at relapse [34]. Nevertheless, survival in ER, LR, and VLR is excellent, with cure rates >97% regardless of the onset of recurrence; therefore, stratification according to ER, LR, and VLR for men with relapse after localised disease is probably clinically irrelevant [34].

By contrast, after first-line chemotherapy, men with LR/VLR more commonly present with both elevated STMs and retroperitoneal disease in comparison to men with ER [52]. These patients often show a distinct tumour biology with a higher risk of malignant transformation [53]. In addition, response to salvage chemotherapy is limited, leading to poor survival [36]. Therefore, surgery instead of chemotherapy is often the salvage approach recommended for men with resectable LR/VLR after first-line chemotherapy [36]. It could therefore be argued that after first-line or salvage chemotherapy, follow-up should be lifelong to identify LR/VLR and initiate salvage surgery within a curative window.

## 4 Conclusions

We summarised published follow-up protocols and discuss current and future developments to personalise the oncological follow-up for patients with tGCT. Functional follow-up, including screening for late toxicities, represents another important aspect of follow-up in this patient population.

  ***Author contributions***: Christian Daniel Fankhauser had full access to all the data in the study and takes responsibility for the integrity of the data and the accuracy of the data analysis.

*Study concept and design*: Fankhauser, Kaufmann, Antonelli.

*Acquisition of data*: Kaufmann, Antonelli.

*Analysis and interpretation of data*: Fankhauser, Kaufmann.

*Drafting of the manuscript*: Fankhauser, Kaufmann.

*Critical revision of the manuscript for important intellectual content*: Fankhauser, Kaufmann, Albers, Cary, Gillessen Sommer, Heidenreich, Oing, Oldenburg, Pierorazio, Stephenson.

*Statistical analysis*: Fankhauser, Kaufmann.

*Obtaining funding*: None.

*Administrative, technical, or material support*: Fankhauser.

*Supervision*: Fankhauser.

*Other*: None.

  ***Financial disclosures:*** Christian Daniel Fankhauser certifies that all conflicts of interest, including specific financial interests and relationships and affiliations relevant to the subject matter or materials discussed in the manuscript (eg, employment/affiliation, grants or funding, consultancies, honoraria, stock ownership or options, expert testimony, royalties, or patents filed, received, or pending), are the following: None.

  ***Funding/Support and role of the sponsor*:** None.

## References

[1] Hanna N.H., Einhorn L.H. (2014). Testicular cancer—discoveries and updates. N Engl J Med.

[2] Kollmannsberger C., Tandstad T., Bedard P.L. (2015). Patterns of relapse in patients with clinical stage I testicular cancer managed with active surveillance. J Clin Oncol.

[3] Tyrrell H.E.J., Church D.N., Joseph J. (2018). Changing practice evaluation—stage 1 seminoma: outcomes with adjuvant treatment versus surveillance: risk factors for recurrence and optimizing follow-up protocols-experience from a supraregional center. Clin Genitourin Cancer.

[4] Kamba T., Kamoto T., Okubo K. (2010). Outcome of different post-orchiectomy management for stage I seminoma: Japanese multi-institutional study including 425 patients. Int J Urol.

[5] Aparicio J., García Del Muro X., Maroto P. (2021). Patterns of relapse and treatment outcome after active surveillance or adjuvant carboplatin for stage I seminoma: a retrospective study of the Spanish Germ Cell Cancer Group. Clin Transl Oncol.

[6] Daugaard G., Petersen P.M., Rørth M. (2003). Surveillance in stage I testicular cancer. APMIS.

[7] Mortensen M.S., Lauritsen J., Gundgaard M.G. (2014). A nationwide cohort study of stage I seminoma patients followed on a surveillance program. Eur Urol.

[8] Tandstad T., Ståhl O., Dahl O. (2016). Treatment of stage I seminoma, with one course of adjuvant carboplatin or surveillance, risk-adapted recommendations implementing patient autonomy: a report from the Swedish and Norwegian Testicular Cancer Group (SWENOTECA). Ann Oncol.

[9] Dieckmann K.P., Dralle-Filiz I., Matthies C. (2016). Testicular seminoma clinical stage 1: treatment outcome on a routine care level. J Cancer Res Clin Oncol.

[10] Chau C., Cathomas R., Wheater M. (2015). Treatment outcome and patterns of relapse following adjuvant carboplatin for stage I testicular seminomatous germ-cell tumour: results from a 17-year UK experience. Ann Oncol.

[11] Joffe J.K., Cafferty F.H., Murphy L. (2022). Imaging modality and frequency in surveillance of stage I seminoma testicular cancer: results from a randomized, phase III, noninferiority trial (TRISST). J Clin Oncol.

[12] White P.M., Howard G.C., Best J.J., Wright A.R. (1997). The role of computed tomographic examination of the pelvis in the management of testicular germ cell tumours. Clin Radiol.

[13] Sadow C.A., Maurer A.N., Prevedello L.M., Sweeney C.J., Silverman S.G. (2016). CT restaging of testicular germ cell tumors: the incidence of isolated pelvic metastases. Eur J Radiol.

[14] Boormans J.L., Mayor de Castro J., Marconi L. (2018). Testicular tumour size and rete testis invasion as prognostic factors for the risk of relapse of clinical stage I seminoma testis patients under surveillance: a systematic review by the Testicular Cancer Guidelines Panel. Eur Urol.

[15] Fankhauser C.D., Christiansen A.J., Rothermundt C. (2022). Detection of recurrences using serum miR-371a-3p during active surveillance in men with stage I testicular germ cell tumours. Br J Cancer.

[16] Daugaard G., Gundgaard M.G., Mortensen M.S. (2014). Surveillance for stage I nonseminoma testicular cancer: outcomes and long-term follow-up in a population-based cohort. J Clin Oncol.

[17] Tandstad T., Cohn-Cedermark G., Dahl O. (2010). Long-term follow-up after risk-adapted treatment in clinical stage 1 (CS1) nonseminomatous germ-cell testicular cancer (NSGCT) implementing adjuvant CVB chemotherapy. A SWENOTECA study. Ann Oncol.

[18] Gels M.E., Hoekstra H.J., Sleijfer D.T. (1995). Detection of recurrence in patients with clinical stage I nonseminomatous testicular germ cell tumors and consequences for further follow-up: a single-center 10-year experience. J Clin Oncol.

[19] Larsen S.K.A., Agerbæk M., Jurik A.G., Pedersen E.M. (2020). Ten years of experience with MRI follow-up of testicular cancer stage I: a retrospective study and an MRI protocol with DWI. Acta Oncol.

[20] Rustin G.J., Mead G.M., Stenning S.P. (2007). Randomized trial of two or five computed tomography scans in the surveillance of patients with stage I nonseminomatous germ cell tumors of the testis: Medical Research Council Trial TE08, ISRCTN56475197—the National Cancer Research Institute Testis Cancer Clinical Studies Group. J Clin Oncol.

[21] De La Pena H., Sharma A., Glicksman C. (2017). No longer any role for routine follow-up chest x-rays in men with stage I germ cell cancer. Eur J Cancer.

[22] Harvey M.L., Geldart T.R., Duell R., Mead G.M., Tung K. (2002). Routine computerised tomographic scans of the thorax in surveillance of stage I testicular non-seminomatous germ-cell cancer—a necessary risk?. Ann Oncol.

[23] White P.M., Adamson D.J., Howard G.C., Wright A.R. (1999). Imaging of the thorax in the management of germ cell testicular tumours. Clin Radiol.

[24] Sharir S., Jewett M.A., Sturgeon J.F. (1999). Progression detection of stage I nonseminomatous testis cancer on surveillance: implications for the followup protocol. J Urol.

[25] Gariscsak P.J., Anson-Cartwright L., Atenafu E.G. (2022). Safety of minimizing intensity of follow-up on active surveillance for clinical stage I testicular germ cell tumors. Eur Urol Open Sci.

[26] Nichols C.R., Catalano P.J., Crawford E.D., Vogelzang N.J., Einhorn L.H., Loehrer P.J. (1998). Randomized comparison of cisplatin and etoposide and either bleomycin or ifosfamide in treatment of advanced disseminated germ cell tumors: an Eastern Cooperative Oncology Group, Southwest Oncology Group, and Cancer and Leukemia Group B Study. J Clin Oncol.

[27] Albers P., Albrecht W., Algaba F. (2015). Guidelines on testicular cancer: 2015 update. Eur Urol.

[28] Cathomas R., Klingbiel D., Bernard B. (2018). Questioning the value of fluorodeoxyglucose positron emission tomography for residual lesions after chemotherapy for metastatic seminoma: results of an International Global Germ Cell Cancer Group registry. J Clin Oncol.

[29] Bachner M., Loriot Y., Gross-Goupil M. (2012). 2–^18^Fluoro-deoxy-D-glucose positron emission tomography (FDG-PET) for postchemotherapy seminoma residual lesions: a retrospective validation of the SEMPET trial. Ann Oncol.

[30] Beyer J., Collette L., Sauvé N. (2021). Survival and new prognosticators in metastatic seminoma: results from the IGCCCG-Update Consortium. J Clin Oncol.

[31] Tandstad T., Smaaland R., Solberg A. (2011). Management of seminomatous testicular cancer: a binational prospective population-based study from the Swedish Norwegian Testicular Cancer Study Group. J Clin Oncol.

[32] Domont J., Massard C., Patrikidou A. (2013). A risk-adapted strategy of radiotherapy or cisplatin-based chemotherapy in stage II seminoma. Urol Oncol.

[33] Suzuki K., Hoshi S., Orikasa S. (2001). Recurrence pattern of metastatic testicular cancers after chemotherapy. Tohoku J Exp Med.

[34] Mortensen M.S., Lauritsen J., Kier M.G. (2016). Late relapses in stage I testicular cancer patients on surveillance. Eur Urol.

[35] Geldart T.R., Gale J., McKendrick J., Kirby J., Mead G. (2006). Late relapse of metastatic testicular nonseminomatous germ cell cancer: surgery is needed for cure. BJU Int.

[36] Ronnen E.A., Kondagunta G.V., Bacik J. (2005). Incidence of late-relapse germ cell tumor and outcome to salvage chemotherapy. J Clin Oncol.

[37] Oldenburg J., Alfsen G.C., Waehre H., Fosså S.D. (2006). Late recurrences of germ cell malignancies: a population-based experience over three decades. Br J Cancer.

[38] Livsey J.E., Taylor B., Mobarek N., Cooper R.A., Carrington B., Logue J.P. (2001). Patterns of relapse following radiotherapy for stage I seminoma of the testis: implications for follow-up. Clin Oncol.

[39] Lehnich A.T., Rusner C., Chodick G., Katz R., Sella T., Stang A. (2019). Actual frequency of imaging during follow-up of testicular cancer in Israel-a comparison with the guidelines. Eur Radiol.

[40] Bäuerle T., Saake M., Uder M. (2021). Gadolinium-based contrast agents: what we learned from acute adverse events, nephrogenic systemic fibrosis and brain retention. RoFo.

[41] Endo T., Kawai K., Kamba T. (2014). Risk factors for loss to follow-up during active surveillance of patients with stage I seminoma. Jpn J Clin Oncol.

[42] Gyawali B., Griffiths R., Robinson A.G., McInnes M.D.F., Bedard P.L., Booth C.M. (2022). Use of imaging for active surveillance in testicular cancer: Is real-world practice concordant with guidelines?. Can Urol Assoc J.

[43] Rusner C., Stang A., Dieckmann K.P., Friedel H. (2013). Frequency of computed tomography examinations in the follow-up care of testicular cancer patients – an evaluation of patterns of care in Germany. Onkologie.

[44] Sharma P., Dhillon J., Agarwal G., Zargar-Shoshtari K., Sexton W.J. (2015). Disparities in interpretation of primary testicular germ cell tumor pathology. Am J Clin Pathol.

[45] Harari S.E., Sassoon D.J., Priemer D.S. (2017). Testicular cancer: the usage of central review for pathology diagnosis of orchiectomy specimens. Urol Oncol.

[46] Nason G.J., Sweet J., Landoni L. (2020). Discrepancy in pathology reports upon second review of radical orchiectomy specimens for testicular germ cell tumors. Can Urol Assoc J.

[47] Fankhauser CD, Nuño MM, Murray MJ, Frazier L, Bagrodia A. Circulating microRNAs for detection of germ cell tumours: a narrative review. Eur Urol Focus. In press. 10.1016/j.euf.2022.04.008. 35537936

[48] Dieckmann K.P., Radtke A., Geczi L. (2019). Serum levels of microRNA-371a-3p (M371 test) as a new biomarker of testicular germ cell tumors: results of a prospective multicentric study. J Clin Oncol.

[49] Terbuch A., Adiprasito J.B., Stiegelbauer V. (2018). miR-371a-3p serum levels are increased in recurrence of testicular germ cell tumor patients. Int J Mol Sci.

[50] Lafin J.T., Singla N., Woldu S.L. (2020). Serum microRNA-371a-3p levels predict viable germ cell tumor in chemotherapy-naïve patients undergoing retroperitoneal lymph node dissection. Eur Urol.

[51] Lobo J., Leão R., Gillis A.J. (2021). Utility of serum miR-371a-3p in predicting relapse on surveillance in patients with clinical stage I testicular germ cell cancer. Eur Urol Oncol.

[52] O’Shaughnessy M.J., Feldman D.R., Carver B.S., Sheinfeld J. (2015). Late relapse of testicular germ cell tumors. Urol Clin North Am.

[53] Sharma A., Alifrangis C., Milic M. (2019). Somatic transformation in metastatic testicular germ cell tumours – a different disease entity. Anticancer Res.

