# Supplementary material for: Oncological Follow-up Strategies for Testicular Germ Cell Tumours: A Narrative Review
Source: Eur Urol Open Sci. 2022 Sep 7;44:142–9. doi: 10.1016/j.euros.2022.08.014 (PMC9465095; doi:10.1016/j.euros.2022.08.014)
Supplement: Supplementary Fig. 1 [file mmc1.docx]

Supplementary figure 1: flowchart of systematic review search
