# Supplementary material for: Oncological Follow-up Strategies for Testicular Germ Cell Tumours: A Narrative Review
Source: Eur Urol Open Sci. 2022 Sep 7;44:142–9. doi: 10.1016/j.euros.2022.08.014 (PMC9465095; doi:10.1016/j.euros.2022.08.014)
Supplement: Supplementary Table 1 [file mmc2.docx]

**Supplementary Table 1** Included studies for review

| **title** | **author** | **year of publication** | **country** | **sample size** | **observation period/ time of treatment or diagnosis** | **clinicopathological factors** | **prospective/**  **retrospective** |
| --- | --- | --- | --- | --- | --- | --- | --- |
| Imaging Modality and Frequency in Surveillance of Stage I Seminoma Testicular Cancer: Results From a Randomized, Phase III, Noninferiority Trial (TRISST) | Joffe, J. K. | 2022 | United Kingdom | 669 | 2008-2014 | Stage I Seminoma | P |
| Use of imaging for active surveillance in testicular cancer: Is real-world practice concordant with guidelines? | Gyawali, B. | 2022 | Canada | 1676 | 2000-2010 | Stage I (NSGCT + Seminoma) | r |
| Safety of minimizing intensity of follow-up on active surveillance for clinical stage I testicular germ cell tumors | Gariscsak, P. J. | 2022 | Canada | 1583 | 1980-2021 | Stage I (NSGCT + Seminoma) | r |
| Patterns of relapse and treatment outcome after active surveillance or adjuvant carboplatin for stage I seminoma: a retrospective study of the Spanish Germ Cell Cancer Group | Aparicio, J. | 2021 | Spain | 879 | 1994-2015 | Stage I Seminoma | r |
| Ten years of experience with MRI follow-up of testicular cancer stage I: a retrospective study and an MRI protocol with DWI | Larsen, S. K. A. | 2020 | Denmark | 759 | 2008-2018 | Stage I (NSGCT + Seminoma) | r |
| Actual frequency of imaging during follow-up of testicular cancer in Israel-a comparison with the guidelines | Lehnich, A. T. | 2019 | Israel | 226 | 2003-2007 | Stage I (nearly all) (NSGCT + Seminoma) | r |
| Detection of Relapse by Low-dose Computed Tomography During Surveillance in Stage I Testicular Germ Cell Tumours | Chung, P. | 2019 | Canada | 256 | ? | Stage I (NSGCT + Seminoma) | P |
| Changing Practice Evaluation-Stage 1 Seminoma: Outcomes With Adjuvant Treatment Versus Surveillance: Risk Factors for Recurrence and Optimizing Follow-up Protocols-Experience From a Supraregional Center | Tyrrell, H. E. J. | 2018 | United Kingdom | 501 | 2004-2016 | Stage I Seminoma | r |
| No longer any role for routine follow-up chest x-rays in men with stage I germ cell cancer | De La Pena, H. | 2017 | United Kingdom | 1447 | 2003-2015 | Stage I (NSGCT + Seminoma) | r |
| Treatment outcome and patterns of relapse following adjuvant carboplatin for stage I testicular seminomatous germ-cell tumour: results from a 17-year UK experience | Chau, C. | 2015 | United Kingdom | 517 | 1996-2013 | Stage I Seminoma | r |
| Patterns of relapse in patients with clinical stage I testicular cancer managed with active surveillance | Kollmannsberger, C. | 2015 | Canada, Norway, Sweden | 2483 | 1998-2010 | Stage I (NSGCT + Seminoma) | r |
| Surveillance for stage I nonseminoma testicular cancer: outcomes and long-term follow-up in a population-based cohort | Daugaard, G. | 2014 | Denmark | 1226 | 1984-2007 | Stage I NSGCT | r |
| Frequency of computed tomography examinations in the follow-up care of testicular cancer patients - an evaluation of patterns of care in Germany | Rusner, C. | 2013 | Germany | 139 | 2005-2006 | not known | r |
| Detection of second malignancies during long-term follow-up of testicular cancer survivors | Buchler, T. | 2011 | Czech Republic | 1057 | 1993-2001 | TGCT | r |
| Randomized trials in 2466 patients with stage I seminoma: patterns of relapse and follow-up | Mead, G. M. | 2011 | United Kingdom | 2466 | 1989-2001 | Stage I Seminoma | P |
| Long-term follow-up after risk-adapted treatment in clinical stage 1 (CS1) nonseminomatous germ-cell testicular cancer (NSGCT) implementing adjuvant CVB chemotherapy. A SWENOTECA study | Tandstad, T. | 2010 | Norway | 232 | 1995-1998 | Stage I NSGCT | P |
| Outcome of different post-orchiectomy management for stage I seminoma: Japanese multi-institutional study including 425 patients | Kamba, T. | 2010 | Japan | 425 | 1986-2006 | Stage I Seminoma | r |
| What is the value of routine follow-up in stage I seminoma after paraaortic radiotherapy?: an analysis of the German Testicular Cancer Study Group (GTCSG) in 675 prospectively followed patients | Clasen, J. | 2009 | Germany | 675 | 1991-1994 | Stage I Seminoma | P |
| Randomized trial of two or five computed tomography scans in the surveillance of patients with stage I nonseminomatous germ cell tumors of the testis: Medical Research Council Trial TE08, ISRCTN56475197--the National Cancer Research Institute Testis Cancer Clinical Studies Group | Rustin, G. J. | 2007 | United Kingdom, Norway, Australia, and New Zealand | 414 | 1998-2013 | Stage I NSGCT | P |
| The utility of lactate dehydrogenase in the follow-up of testicular germ cell tumours | Venkitaraman, R. | 2007 | United Kingdom | 499 | 2004-2005 | TGCT | r |
| Long-term follow-up of Anglian Germ Cell Cancer Group surveillance versus patients with Stage 1 nonseminoma treated with adjuvant chemotherapy | Oliver, R. T. | 2004 | United Kingdom | 382 | 1978-2000 | Stage I NSGCT | r |
| Efficacy of routine follow-up after first-line treatment for testicular cancer | Spermon, J. R. | 2004 | Netherlands | 505 | 1982-2000 | TGCT | r |
| Follow-up of clinical stage I testicular cancer patients: cost and risk benefit considerations | Kakehi, Y. | 2002 | Japan | 119 | 1990-1997 | Stage I (NSGCT + Seminoma) | r |
| Routine computerised tomographic scans of the thorax in surveillance of stage I testicular non-seminomatous germ-cell cancer--a necessary risk? | Harvey, M. L. | 2002 | United Kingdom | 168 | 1986-1998 | Stage I NSGCT | r |
| Patterns of relapse following radiotherapy for stage I seminoma of the testis: implications for follow-up | Livsey, J. E. | 2001 | United Kingdom | 409 | 1988-1997 | Stage I Seminoma | r |
| Detection of recurrence in patients with clinical stage I nonseminomatous testicular germ cell tumors and consequences for further follow-up: a single-center 10-year experience | Gels, M. E. | 1995 | Netherlands | 154 | 1982-1992 | Stage I NSGCT | r |
| Orchidectomy followed by radiotherapy in 176 stage I and II testicular seminoma patients: benefits of a 10-year follow-up study | Bayens, Y. C. | 1992 | Netherlands | 176 | 1975-1985 | Stage I + II Seminoma | r |
| The value of repeated chest radiographs in the follow-up of patients with germ cell testicular tumours | Dunn, W. K. | 1991 | United Kingdom | 183 | 1981-1988 | Stage I Seminoma + Teratoma (all stages) | r |
| Early clinical stages of nonseminomatous testis cancer. Evaluation of the primary treatment and follow-up procedures of the SWENOTECA project | Klepp, O. | 1991 | Norway, Sweden | 345 | 1981-1986 | NSGCT (all stages) | P |
